# Muscle Synergy of the Underwater Undulatory Swimming in Elite Male Swimmers

**DOI:** 10.3389/fspor.2020.00062

**Published:** 2020-06-03

**Authors:** Yuiko Matsuura, Naoto Matsunaga, Satoshi Iizuka, Hiroshi Akuzawa, Koji Kaneoka

**Affiliations:** ^1^Faculty of Sport Sciences, Waseda University, Tokorozawa, Japan; ^2^General Education Core Curriculum Division, Seigakuin University, Ageo, Japan

**Keywords:** EMG, muscle synergies, NMF, swimming, underwater undulatory swimming, elite swimmers

## Abstract

Improving the performance of underwater undulatory swimming (UUS) improves swimming time, so it is important to identify the pattern of muscle coordination in swimmers with fast UUS. This study aimed to identify muscular coordination in the trunk and lower limb during UUS in elite swimmers. Nine swimmers (aged 20 ± 2 years; height, 1.74 ± 0.03 m; weight, 73.0 ± 4.4 kg) participated in this study. Measurements were taken by electromyography of eight muscles: rectus abdominis (RA), internal abdominal muscle (IO), rectus femoris (RF), erector spinae (ES), multifidus (MF), tibialis anterior (TA), and thigh biceps (BF), and gastrocnemius (GS). For evaluation of muscle coordination, “muscle synergy” and “activation coefficient” were calculated using non-negative matrix factorization from electromyographic data. Kick frequency, kick amplitude, swim velocity, and kinematics of the pelvis were also calculated. Kick cycle was divided into two kick phases: downward kick (from the highest toe vertical coordinate to the lowest point) and upward kick (from the lowest point to the highest point). Kick frequency, kick amplitude, and swimming velocity were 1.9 ± 0.3 Hz, 0.45 ± 0.6 m, and 1.8 ± 0.2 m·s ^−1^, respectively. The maximum backward pelvic tilt was 94.4 ± 4.5° and the minimum (forward) was 90.8 ± 5.7°. Three muscle synergy values were extracted from each swimmer during UUS: those involved in the transition from upward kick to downward kick (Synergy 1), downward kick (Synergy 2), and upward kick (Synergy 3). Synergy 1 involved mainly the RF, IO, and RA, which were activated during the turn from the upward to the downward phase. Synergy 2 involved mainly the MF, ES, and TA in the downward kick. Synergy 3 corresponded to the coordination of the BF and GS, which were active in the upward kick. In UUS by elite swimmers, both the upward kick and downward kick followed the trunk muscles involved in the pelvic forward–backward tilt movement, and lower limb muscles were activated. Muscle coordination based on pelvic forward-backward tilt during UUS is expected to contribute to the coaching field for elite swimmer development.

## Introduction

Underwater undulatory swimming (UUS), also called dolphin kick or a submerged propulsion technique used in competitive swimming, has gained prominence as a “fifth stroke” (Collard and Oboeuf, 2009). There are four well-accepted swimming techniques (butterfly, backstroke, breaststroke, and front crawl), and the UUS is a phase during the start and turn race segments (Pereira et al., 2015). In these race events, the present international rules allow swimmers to perform UUS until 15 m after the start and turns for butterfly, front crawl, and backstroke swimming, and only one dolphin kick is allowed for breaststroke. Therefore, improving the performance of UUS is likely to improve the time during swimming races (Veiga et al., 2016). Previous studies reported that an increased kick frequency improves the UUS speed (von Loebbecke et al., 2009; Shimojo et al., 2014; Connaboy et al., 2016). These studies all performed kinematics analyses. Recently, Shimojo et al. reported the visualization of three-dimensional flow in the wake region during human UUS in a water flume (Shimojo et al., 2019a). The USS is currently popular research topic.

In contrast, few studies have examined UUS using electromyography (EMG) (Barthels and Adrian, 1971; Martens et al., 2015b; Yamakawa et al., 2017). Few have evaluated the co-contractions of the main movement and antagonist muscles (Jammes et al., 2010; Yamakawa et al., 2017). Although this evaluation method could assess the coordination between agonist and antagonist muscles, it was difficult to evaluate the coordination among multiple muscles. Recently, the non-negative matrix factorization (NMF) analysis, which is based on Bernstein's concept of muscle synergy (NA 1967), has been used to evaluate all muscle coordination. The NMF analysis requires EMG data separated into two: “muscle synergy” and “activation coefficient.” Muscle synergy indicates the coordination of muscles, while the activation coefficient indicates the activation timing of the muscle synergy (Ting and McKay, 2007; Chvatal and Ting, 2013). The combination of these influences is called a “synergy,” and a theory suggests that human movement is performed by various synergies. Sports activity has also been analyzed using NMF, although related research is limited (Hug et al., 2010; Turpin et al., 2011; Taborri et al., 2018). In swimming, only breaststroke has been subjected to a muscle synergy analysis (Vaz et al., 2016).

If muscular coordination during UUS of elite swimmers becomes clear, we believe that it will contribute to the coaching field on how to become elite swimmers. This study aimed to identify the muscular coordination in the trunk and lower limb during UUS in elite swimmers. We hypothesized that two synergies related to the upward and downward kicks would be extracted because UUS involves a two-way periodic movement of upward and downward kicks in kinematics data (Hermens et al., 1999; Cohen et al., 2012).

## Materials and Methods

### Subjects

Nine men, with a mean ± standard deviation age of 20 ± 2 years, height of 1.74 ± 0.03 m, and weight of 73.0 ± 4.4 kg, participated in this study. They practiced with a collegiate swimming team 9 times per week and had received a mean FINA point value of 821.1 ± 68.2 points for their best records in their individual special styles. None of the subjects specialized in breaststroke. The subjects were swimmers specialized in the butterfly, front crawl, and individual medley styles using UUS. Additionally, these subjects were elite swimmers ranked in the top 16 in Japan, and one had received a medal in a butterfly event during the Rio Olympics. Swimmers with a history of lower limb disorders, neurological disorders, or lower limb surgery were excluded.

The participants were made fully aware of the risks, benefits, and stresses of the study, and their written informed consent was obtained. The study was conducted in accordance with the Helsinki Declaration and approved by the University's Research Ethics Committee (2016-267).

### Data Measurement

Experiments were conducted in a 50-m indoor pool in our university. Two cameras (high-speed 1394, DKH Inc., Japan) filmed the sagittal movements of the swimmers and recorded video through underwater windows at a 200-Hz sampling rate. For the two-dimensional (2D) analysis, 13 points were marked with wireless LED markers (Kirameki, Nobby Tech Inc., Tokyo, Japan) on the right side of each participant. These anatomical landmarks corresponded to the tragus, superior margin of the sternum, acromion, lateral epicondyle of the humerus, inferior end of the 10th rib, styloid process, anterior superior iliac spine (ASIS), posterior superior iliac spine (PSIS), greater trochanter, lateral epicondyle of the femur, lateral malleolus, calcaneus, and epiphysis of the fifth metatarsal (toe).

Muscle activity was measured using a wireless EMG system (Biolog2, S & ME Inc., Tokyo, Japan). Measurements were taken on the following eight muscles on the right side of each swimmer: rectus abdominis (RA), internal abdominal muscle (IO), rectus femoris (RF), erector spinae (ES), multifidus (MF), tibialis anterior (TA), thigh biceps (BF), and gastrocnemius (GS). Measurements were taken according to previous studies on UUS (Yamakawa et al., 2017). The electrodes were placed as follows: RA, 3 cm lateral to the umbilicus (Okubo et al., 2010b); IO, ~1 cm medial and inferior to the ASIS (Ng et al., 1998); RF, on the belly of the muscle corresponding to the central point between the ASIS and the upper margin of the patella (Hermens et al., 1999); ES, 3 cm lateral to the L4 spinous process (Okubo et al., 2010b); MF, 2 cm lateral to the lumbar (L) 5 spinous process (Okubo et al., 2010a); TA, at 1/3 on the line between the tip of the fibula and the tip of the medial malleolus (Hermens et al., 1999); BF, at the midpoint of the line between the ischial tuberosity and the lateral epicondyle of the tibia (Hermens et al., 1999); and GS, on the largest bulge of the medial head of the GS muscle (Hermens et al., 1999).

Before surface electrode attachment, the skin was scrubbed with a skin abrasive and alcohol to reduce the impedance to a level <2 k Ω. Pairs of disposable Ag/AgCl surface electrodes (BlueSensor N-00-S, METS Co., Japan) were attached parallel to the muscle fibers. The sampling frequency was set at 1,000 Hz. To normalize the EMG data, a maximum voluntary contraction (MVC) test was performed on each muscle before the measurement in water.

The MVC of the RA was obtained while the subject performed a partial sit-up with the knees flexed and manual resistance applied. The MVC for the IO was determined by performing the following: flexion and rotation of the trunk to the right, while resistance was applied at the shoulders, with extension and rotation of the trunk to the left. To obtain the MVC of the RF, the subject sat on a chair with their hips and knees flexed at 90° and performed knee extensions. Resistance was applied at the shank in the direction of knee flexion and subsequently at the shank in the direction of knee extension. For the ES and MF, the MVC task comprised a trunk extension performed in the prone position without leg movement, with manual resistance applied to the upper thoracic area. For the TA, the subject sat on a floor with the hips flexed and knees extended to 0° and performed ankle dorsiflexion. Resistance was applied to the soles of the feet in the direction of ankle extension. The MVC of the BF was obtained with the subject in a prone position and a consistent knee flexion angle of 45°. The MVC of the GS was obtained while the subject stood on one leg and maintained maximum ankle plantarflexion; at the same time, the examiner applied a vertical downward force to the subject's shoulder. Manual resistance was increased gradually until the subject's limit was reached and held for 3 s. EMG data were normalized as a percentage of the highest RMS amplitude obtained over a 1 s period during MVC tests.

Using the methodology of Kobayashi et al. (2015), the electrodes were covered with water-resistant tape for waterproofing. In the present study, transparent dressing tape (Tegaderm Film Roll, 3M Health Care Inc.) and stretchable adhesive tape (Cover-Roll Stretch, Beiersdorf Inc., Wilton, CT) were used for waterproofing. To synchronize the video and EMG data, a synchronizer (PTS-110, DKH Inc., Japan) was connected to both trigger channels. Video and EMG data were recorded simultaneously.

The experimental protocol was similar to that used by Yamakawa et al. (2017). The experimental task comprised a 15-m prone UUS. Before the task, the swimmers performed a moderate-intensity 1,000-m warm-up swim. After the warm-up session, electromyograms were attached, and MVCs were measured. After measuring all MVCs, the swimmers performed the UUS while swimming at maximal effort from a push-off start until they passed a point 15 m from the starting point. Considering that there is only one race, we conducted one maximum trial in this study. The participants swam ~1.0 m under water to reduce the effect of wave drag (Lyttle et al., 2000). The duration between consecutive time points when the toe was at the highest point during the kick cycle was investigated using the video that was filmed at 8–12 m from the starting point. All variables were taken on three consecutive cycles within the calibrated area.

### Data Analysis

Three UUS cycle measurements were analyzed. A study reported that extracting UUS kinematics is highly reliable to analyze from the average value of three or more cycles as a representative value (Connaboy et al., 2010). In addition, in a previous study that measured EMG of UUS, the average value of three cycles was used as a representative value (Yamakawa et al., 2017). Therefore, three cycles were also analyzed in the present study. Three cycle values were obtained for all variables including kick frequency, kick amplitude, swim velocity, kinematics of the pelvis, and EMG data, and the mean of these values was used for analysis.

#### Kinematics Data

**Figure 1:**
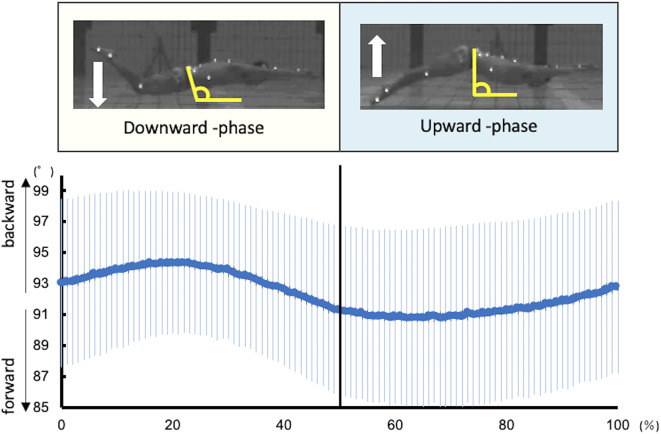
Kinematic data of the pelvic tilt angle during UUS. The angle of the pelvis was defined as the angle between the line connecting the ASIS and PSIS and the horizontal line. The larger the angle, the more backward is the pelvis, and the smaller the angle, the more forward it is.

In this study, one kick cycle started at the highest toe vertical coordinate and ended with the next highest peak thereafter. Each kick cycle was divided into two kick phases: downward kick and upward kick. It was defined with reference to the report by Atkison et al. (2014). The downward kick started from the highest toe vertical coordinate to the lowest point, and an upward kick started from the lowest point to the highest point. The kick frequency and amplitude were calculated from the toe coordinates. Kick frequency was defined as the reciprocal of the duration of one kick cycle. Kick amplitude was defined as the vertical distance between the highest and lowest vertical toe peaks during one kick cycle. The inclination of the pelvis was defined as the angle between the line connecting the ASIS and PSIS and the horizontal line (Figure 1).

#### EMG Data

The recorded EMG data were analyzed using biomedical information software (BIMUTAS-Video, Kissei Comtec Co., Ltd., Japan). The raw data were band-pass filtered (4th order Butterworth) between 20 and 450 Hz and full wave rectified. EMG data were then normalized relative to the muscle's associated MVC data and interpolated to 101 time points. A custom MATLAB (MATLAB R2018, MathWorks, Inc., Natick, MA) code was used to the linear envelope and NMF. For the linear envelope, we used a Hibert filter with a length of 10, and NMF was performed to extract modules as described by Lee and Seung (1999) using the following formulas:

$$\begin{array}{l} \text{ E=WC+e}\ldots \text{formula}\;\text{1}\\ \min{\left\|\text{E-WC}\right\|}_{\text{FRO}}\\ \text{W}>\text{0}\\ \text{C}>\text{0} \end{array}$$

where E is a p-by-n initial matrix, “p” is the number of muscles, and n is the number of time points. The initial matrix comprised normalized EMG data and consisted of a mean of three cycles for each of the eight muscles; therefore, E was a matrix with eight rows and 101 columns. W represent a p-by-s matrix and “s” is the number of synergies and represents muscle synergy. C is an s-by-n matrix that represents the activation coefficient, and “e” is a p-by-n residual error matrix. Formula 2 indicates that matrix “e,” calculated using formula 1, reaches a minimum. W is a vector and therefore is written as $\overset{⇀}{W}$ when calculated. For each subject, we iterated the analysis by varying the number of synergies between 1 and 8 and then selected the least number of synergies fulfilling global variance accounted for (VAF) (Torres-Oviedo et al., 2006; Hug et al., 2010). VAF is defined as 100 × the coefficient of determination from the uncentered Pearson correlation coefficient (Tresch et al., 2006). We defined the standard as the global VAF > 90% and local VAF > 75%.

The global and local VAF were calculated using formulas 3 and 4.

$$\begin{array}{l} \text{Global VAF}=(\text{1-}\frac{\sum_{\text{i=1}}^{\text{p}}\sum_{\text{j=1}}^{\text{n}}\;{\left({\text{e}}_{\text{i},\text{j}}\right)}^{\text{2}}}{\sum_{\text{i=1}}^{\text{p}}\sum_{\text{j=1}}^{\text{n}}\;{\left({\text{E}}_{\text{i},\text{j}}\right)}^{\text{2}}})\times \text{100}[\%]\\ \;\ldots \text{formula}\;\text{3} \end{array}$$

$$\text{Local VAF }[\text{m}]=(\text{1-}\frac{\sum_{\text{j=1}}^{\text{n}}\;{\left({\text{e}}_{\text{m},\text{j}}\right)}^{\text{2}}}{\sum_{\text{j=1}}^{\text{n}}\;{\left({\text{E}}_{\text{m},\text{j}}\right)}^{\text{2}}})\times \text{100}[\%]\ldots \text{formula}\;\text{4}$$

where “i” ranges from 1 to “p,” “j” ranges from 1 to n, and m represents the muscle. Thus, “i” ranged between 1 and 8 and “j” ranged between 1 and 101 in this study.

## Results

Table 1 presents the kinematic variables during the UUS. Kick frequency, kick amplitude, and swimming velocity were 1.9 ± 0.3 Hz, 0.45 ± 0.6 m, and 1.8 ± 0.2 m·s ^−1^, respectively. The ratio of each phase in one cycle was 48.5 ± 3.6% in the downward kick phase and 51.5 ± 3.6% in the upward kick phase. Table 2 describes the relationship between the number of modules and the global and local VAF. Initially, when two synergies were present, the mean global VAF exceeded 90%, but the mean local VAF did not exceed 75%. However, when three synergies were present, the mean global VAF exceeded 90% and the mean local VAF exceeded 75%. Therefore, the three synergies were obtained during the UUS in this study. None of the swimmers reach the established threshold (global VAF >90 and local >75) when two synergies were applied.

**Table 1:** Kinematic variables measured during the UUS.

**Variables**	**Unit**	
Kick frequency	(Hz)	1.9 ± 0.3
Kick amplitude	(m)	0.45 ± 0.06
Swimming velocity	(m·s^−1^)	1.8 ± 0.2
Downward kick phase	(%)	48.5 ± 3.6
Upward kick phase	(%)	51.5 ± 3.6

*One kick cycle started at the highest toe vertical coordinate and ended with the highest peak thereafter. The kick frequency and amplitude were calculated from the toe coordinates. Kick frequency was defined as the reciprocal of the duration of one kick cycle. Kick amplitude was defined as the vertical distance between the highest and lowest vertical toe peaks during one kick cycle. The downward kick started from the highest toe vertical coordinate to the lowest point, and an upward kick started from the lowest point to the highest point. Downward and Upward kick phase was the ratio of each phase to one cycle*.

**Table 2:** Relationship between the number of synergies and the global and local VAF.

		**Number of synergies = 2**	**Number of synergies = 3**
Global VAF (%)		92.0 ± 8.8	92.0 ± 0.9
Local VAF (%)	RA	74.9 ± 17.7	83.8 ± 13.4
	IO	75.6 ± 19.2	84.7 ± 15.9
	RF	87.0 ± 12.1	96.4 ± 6.1
	ES	66.6 ± 23.4	94.6 ± 7.2
	MF	82.3 ± 18.6	95.8 ± 3.4
	TA	72.0 ± 22.4	96.0 ± 3.2
	BF	75.0 ± 23.9	93.6 ± 7.4
	GS	73.2 ± 20.7	96.4 ± 3.0

*The number of synergies was set as the lowest number at which the global VAF exceeded 90% and the local VAF exceeded 75%. When the number of synergies were 3, the global VAF exceeded 90%, and the local VAF exceeded 75% for all muscles, so it was determined that there were three synergies during the UUS. BF, biceps femoris; ES, erector spinae; GS, gastrocnemius; IO, internal oblique; MF, multifidus; RA, rectus abdominis; RF, rectus femoris; TA, tibialis anterior; VAF, variance accounted for*.

**Figure 2:**
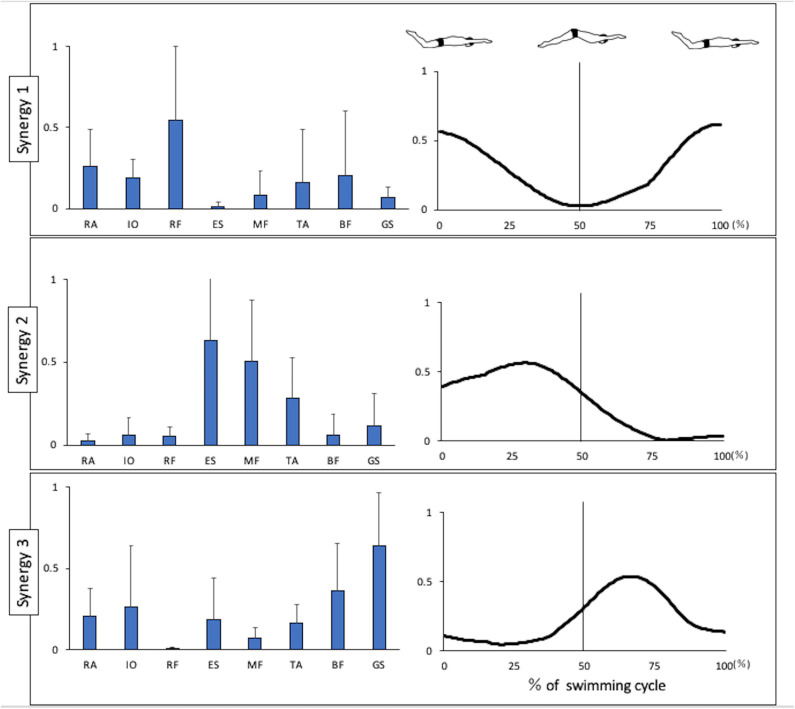
Synergies extracted during UUS. Muscle synergy vectors are shown at the left side of the figure aligned to the corresponding activation coefficient. The synergy activation coefficients are shown in the right side of the figure by synergy. BF, biceps femoris; ES, erector spinae; GS, gastrocnemius; IO, internal oblique; MF, multifidus; RA, rectus abdominis; RF, rectus femoris; TA, tibialis anterior.

Figure 1 shows the kinematics data of the pelvis tilting forward and backward. The maximum range of pelvic backward tilting was 94.4 ± 4.5° at 22% of the UUS cycle. The minimum range of pelvic forward tilting was 90.8 ± 5.7° at 59% of the UUS cycle. Figure 2 depicts the extracted modules of the UUS and the kicks performed by all subjects. Synergy 1 mainly reflected the RF, IO, and RA activities. The activation coefficient of Synergy 1 exceeded 0.5 in 0–10% and 88–100%, and the peak was 99% of the UUS cycle. This phase was defined as the transition from the upward kick to the downward kick. Data from Synergy 2 mainly reflected the MF, ES, and TA activities during the downward kick phase. The peak of the activation coefficient of Synergy 2 was at 29% of the UUS cycle. Synergy 3 mainly reflected the GS and BF activity during the upward kick phase. The peak of the activation coefficient of Synergy 3 was at 66% of the UUS cycle.

**Figure 3:**
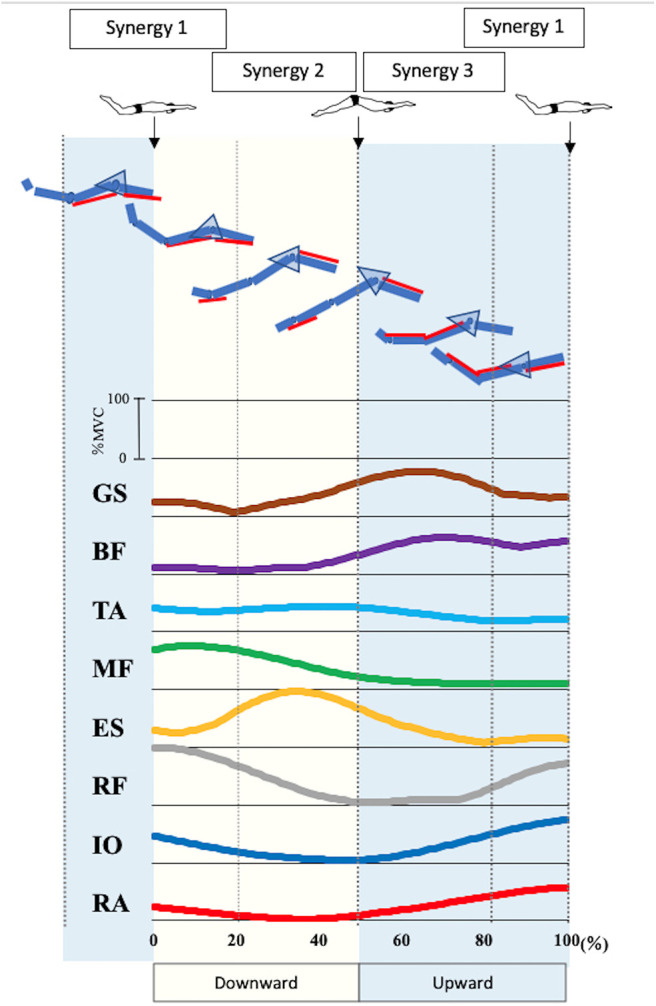
Electromyographic data of each muscle and involvement of in each muscle in the synergies during UUS. The figure shows the electromyographic data of each muscle involved in the UUS in a mean of all subjects. Synergy 1 was involved in tilting the pelvis from the upward kick to the downward kick with the involvement of the RA, IO, and RF. Synergy 2 was involved in the downward kick, with the involvement of the ES, MF and TA. Synergy 3 was involved in the upward kick, with the involvement of the BF and GS. From top to bottom GS, gastrocnemius; BF, biceps femoris; TA, tibialis anterior; MF, multifidus; ES, erector spinae; RF, rectus femoris; IO, internal oblique; RA, rectus abdominis.

Figure 3 presents the EMG data of each muscle during UUS. The IO activation exceeded 50%MVC in 82–100%, and the peak was 100%. The RA activation exceeded 50%MVC in 88–100%, and the peak was 100%. These phases appeared later in the upward kick. The RF activation exceeded 50%MVC in 89–100% and 0–27%, and the peak was 0% of the UUS cycle. The RF became active later in the upward kick to early in the downward kick. The ES activation exceeded 50%MVC in 18–55% and the peak was 35% of the UUS cycle, the MF in 0–32%, and the peak was 9% of the UUS cycle. The TA did not exceed 50%MVC, and the peak was 41% of the UUS cycle. The active phases of the ES, MF, and TA were in the downward kick. The BF was active in 58–100% and the peak was 72% of the UUS cycle. The GS was active in 47–81%, and the peak was 64% of the UUS cycle. The active phases of the BF and GS were the upward kick.

## Discussion

This study investigated the modular control of muscles during UUS. Most notably, we determined that three synergies could be determined during this kick. Previously, von Loebbecke et al. (2009) analyzed the motion of the UUS in Olympic-level male swimmers and reported an average velocity of 1.50 ± 0.29 m·s^−1^, a kick frequency of 2.25 ± 0.34 Hz, and a kick amplitude of 0.56 ± 0.10 m. The kinematic results and values obtained in this study were greater than those reported in that previous study. We note that our subjects included an Olympic medalist in a butterfly event, as well as other swimmers known to have high performance levels in the UUS. Previous studies of muscle activity during the UUS have reported that the main movement and antagonist muscles in the trunk and thigh act alternately (Jammes et al., 2010; Yamakawa et al., 2017). In addition, as the UUS involves a two-way periodic movement of upward and downward kicks, we hypothesized that two synergies related to the upward and downward kicks would be extracted (Hermens et al., 1999; Arellano et al., 2002; Cohen et al., 2012). However, we identified three synergies in the UUS in all swimmers.

In this report, we present the EMG data of each muscle during the UUS in Figure 3. Note that the trunk muscles (IO and RA) were active before the transition from the upward to the downward kick, after which the RF remained active. These activities were believed to follow the BF activity, which had been activated in the previous phase. The IO and RA were actively involved in tilting the pelvis backward. When the pelvis was stable, due to the activity of the trunk muscles, we considered that the propulsive force would be generated by activating the RF, which involves the hip flexion and knee extension muscles. The IO and RA remained active up to ~20%, which is the first half of the downward phase and the maximum degree of backward pelvic, and these activities were considered to constitute Synergy 1 (Figure 2) during the transition from the upward to the downward phase. The RF contributes to knee extension and hip flexion, while the RA and IO were activated during trunk flexion to tilt the pelvis backward and contribute to trunk stability.

Therefore, Synergy 1 was involved in tilting the pelvis from the upward kick to the downward kick. Previously, Kaneoka analyzed the muscle activity during flutter kicks and reported that the IO activity was important for the transition from the upward kick to the downward kick (Kaneoka et al., 2015). In both the UUS and flutter kick, the activity of trunk muscles IO and RA is important during this transition phase. Nakashima (2009) conducted a computer simulation analysis and reported that the undulatory trunk movement during the UUS contributed to improvements in both the swimming velocity and propulsion efficiency. Synergy 1 may therefore be an important synergy for improving both the swimming velocity and propulsion efficiency.

Moreover, we evaluated the ES, MF, and TA, which are activated in the downward phase. Kobayashi concluded that the extensor muscles of the trunk were activated during the downward phase (Kobayashi et al., 2016). In the downward phase, the hip joint is flexed and knee joint is extended (Atkison et al., 2014). Furthermore, in this study, the pelvis begins to tilt from backward to forward (Figure 1). The ES and MF, which represent the extension of trunk muscles, were thought to be activated to restore the pelvic position from the backward tilt. TA is more strongly activated after the activation of RF, the knee extension muscle. The TA activity is high at the end of the downward kick. Although there were no kinematic data for the ankle angle in this study, according to previous study, the ankle joint is maximally in plantarflexion at this timing (Shimojo et al., 2019b), and the propulsion is increased (Shimojo et al., 2019a). The activation of the TA is attributed to the propulsive force generated not only by the knee extension, but also by the ankle plantarflexion. Synergy 2 is considered to constitute these muscles. The ES contributes the most in this synergy, while the activities of the MF and TA were also notable. The peak of activation coefficient of Synergy 2 was at 29% of the UUS cycle, which immediately occur after 22% of the UUS cycle when the pelvis forward tilt begins from the backward tilt. Therefore, we theorized that Synergy 2 is involved in bringing the pelvis back from the backward to forward tilt position during downward kick.

Further, we evaluated the BF and GS. These muscles were activated immediately before the upward phase and remained active late into the upward phase. The TA was also active during the first half of the upward phase, although at a lower level than during the previous phase. In the upward phase, the hip joint is extended and knee joint is flexed (Atkison et al., 2014), and plantarflexion and dorsiflexion (Shimojo et al., 2019b). Previous studies demonstrated the co-contraction of the TA and GS, which would be caused by kicking while maintaining ankle plantarflexion against the resistance of the water (Yamakawa et al., 2017). Synergy 3 represents the coordination of the BF and GS. The knee and ankle flexor and extensor muscles were activated during the upward phase (Kobayashi et al., 2016). This synergy, which is activated during the upward kick, is thought to contribute to knee flexion and ankle plantarflexion, and propulsion is generated by kicking the water upward.

Figure 3 summarizes the results of the pelvic kinematics and muscle synergy findings and considers the motor behaviors when the muscles are activated due to each type of synergy during the UUS. Both the upward kick and downward kick followed the trunk muscles involved in the pelvic forward–backward tilt movement, leading to muscle activity in the lower limb. The ability to learn the movement of the UUS relies on the understanding of this pelvic forward–backward tilt movement (Figure 3).

This study has limitations. This study lacked “full-motion analysis” in the 2D motion analysis. Since the pelvis angle was calculated based on global coordinates, neutral pelvis cannot be defined. Whether or not the pelvis is backwards tilting was a relative value. As the UUS involves little rotational motion, an analysis on the sagittal plane is likely sufficient. In addition, since all the subjects were male swimmers, the same trend may not be applied to female swimmers. A previous study found no difference between male and female swimmers with respect to the kinematic variables of the undulatory underwater kick (Arellano et al., 2002; von Loebbecke et al., 2009). Furthermore, it may be possible to generalize the present results to female swimmers.

Moreover, this study evaluated high-performing subjects. Therefore, the results cannot be generalized to other populations, as demonstrated by Vaz et al. (2016) who compared the synergies obtained during breaststroke between elite and beginner swimmers and found that the timing of water propulsion was earlier in beginner swimmers. Further studies of the muscle synergies obtained by beginners during the UUS are needed to increase the body of knowledge about this technique. A follow-up study that replicates the current study with a population of beginning swimmers may further enable an understanding of how this coordination strategy is modified over time to match that observed in elite swimmers. Martens et al. (2015a, 2016) investigated electromyography in front crawl swimming and reported that high level athletes had low intra-individual variability but high inter-individual variability. Based on these, it is necessary to examine the inter-individual variability by level.

## Conclusion

In this study, we analyzed muscle synergies in trunk and lower limb muscles during UUS in elite swimmers and found three synergies: that is, those involved in the transition from upward kick to downward kick, downward kick, and upward kick. In the UUS in elite swimmers, both the upward kick and downward kick followed the trunk muscles involved in the pelvic forward–backward tilt movement, and muscles in the lower limb were activated. Learning of muscle coordination based on pelvic forward–backward tilt during the UUS is expected to contribute to the coaching field on how to become elite swimmers.

## Data Availability Statement

The datasets generated for this study are available on request to the corresponding author.

## Ethics Statement

The studies involving human participants were reviewed and approved by Waseda University. The patients/participants provided their written informed consent to participate in this study.

## Author Contributions

YM created the main conceptual ideas for the paper. All authors contributed to the manuscript writing.

## Conflict of Interest

The authors declare that the research was conducted in the absence of any commercial or financial relationships that could be construed as a potential conflict of interest.
